# The relationship between nutrition, diet, and early childhood caries: A cross-sectional study using 2009 to 2014 NHANES data

**DOI:** 10.1097/MD.0000000000049124

**Published:** 2026-06-05

**Authors:** Li-Shan Ren, Zi-Ying Ji, Shao-Cong Ma, Hui-Ying Li, Xiao-Xiao Zhang, Qi Sun, Tian-Ke Li, Li-Hong Liu

**Affiliations:** a Department of Stomatology, Children’s Hospital of Hebei Province (Hebei Provincial Clinical Research Center for Child Health and Disease), Hebei Medical University, Shijiazhuang, Hebei, China; b Department of Stomatology, The Fourth Affiliated Hospital of Hebei Medical University, Shijiazhuang, Hebei, China.

**Keywords:** calcium intake, early childhood caries, NHANES, nonlinear relationship, nutritional intervention, restricted cubic spline

## Abstract

Early childhood caries (ECC) is a major public health issue influenced by nutritional, behavioral, and sociodemographic factors. However, the independent effects and dose-response patterns of key risk factors remain unclear. This study aimed to analyze the associations between nutritional and dietary factors and ECC using nationally representative data, and to explore the nonlinear relationship between calcium intake and ECC. Data from 2564 children aged 2 to 6 years were obtained from the National Health and Nutrition Examination Survey 2009 to 2014. Weighted chi-square tests compared baseline characteristics between ECC and non-ECC groups. Multivariate logistic regression assessed the effects of age, calcium, fat, vitamin D, and other factors on ECC. Restricted cubic spline regression explored the nonlinear association between calcium intake and ECC. Forest plots and correlation analyses evaluated the strength of these associations. Significant differences were observed between groups in ethnicity, breastfeeding history, age, calcium and fat intake. Caries prevalence was higher in Mexican Americans (29.1%), breastfed children (61.4%), and 6-year-olds (29.4%). Children with ECC had lower calcium intake (838 vs 893 mg) and higher fat intake. Regression analysis showed that ECC risk increased with age (odds ratio = 6.319, *P* < .001). Restricted cubic spline analysis revealed a significant nonlinear relationship between calcium intake and ECC (*P* = .011), with an inflection point at 1000 mg/day. Below this threshold, increased calcium intake was associated with reduced ECC risk; above it, the effect plateaued. Vitamin D was not significantly associated with ECC. This study identifies 1000 mg/day as a potential threshold for calcium intake in reducing ECC risk. Increasing age and insufficient calcium intake are independent risk factors for ECC. These findings support the development of targeted interventions focusing on dietary optimization, oral microbiota regulation, and feeding behavior adjustment in high-risk pediatric populations.

## 1. Introduction

Early childhood caries (ECC) remains a critical public health challenge globally, with severe consequences extending beyond oral health. Children with ECC frequently experience dental pain, impaired masticatory function, and malnutrition due to disrupted nutrient absorption, which may further lead to growth retardation and reduced quality of life.^[1,2]^ Psychologically, ECC-related stigma (e.g., discolored teeth) can impair social adaptability, while untreated caries in early childhood may increase the risk of permanent tooth decay and systemic inflammation in adulthood.^[3–5]^ National Health and Nutrition Examination Survey (NHANES) studies indicate that the prevalence of ECC among 6-year-old children can reach 29.4%, showing a trend of increasing with age, which suggests that the period after 3 years of age may be a critical window for caries prevention and control.^[6,7]^

Modern etiological studies have revealed that ECC is essentially a dynamic process resulting from the combined effects of genetic susceptibility, microbial ecological imbalance, and environmental exposure. Among these factors, dietary nutrition, as a key modifiable environmental factor, participates in the occurrence and development of ECC through the 3-dimensional interaction mechanism of “nutrition-microbe-enamel.”^[8,9]^

Calcium metabolism, a cornerstone of this triad, directly impacts enamel acid resistance by contributing to the hydroxyapatite structure and enhancing salivary remineralization capacity. Children whose daily calcium intake falls below 800 mg face a significantly elevated risk of ECC, ranging from 1.5 to 2 times higher than their counterparts with adequate intake.^[10,11]^ Dietary fat, another component of the triad, may contribute to cariogenic biofilm formation, particularly when associated with high-sugar diets. It can also alter the β-diversity of oral microbiota, with saturated fatty acids showing a positive correlation with the abundance of *Streptococcus mutans*, a key bacterium in caries development.^[12,13]^ In particular, the intake of saturated fatty acids is positively correlated with the abundance of *S. mutans*, which may be a new mechanism underlying its caries-promoting effect.^[14]^ Breastfeeding, while providing protective immunoglobulins and probiotics through breast milk, exhibits a dual effect. Prolonged nighttime feeding beyond 12 months can create a sustained acidic oral environment, fostering the growth of acid-tolerant flora such as *Lactobacillus* and accelerating enamel demineralization.^[10,15]^ This microbial dysbiosis can accelerate enamel demineralization and exert a synergistic effect with dietary factors.^[16]^ Notably, The deficiency in vitamin D, as a regulator of calcium metabolism, its deficiency may indirectly alter the colonization resistance of oral microorganisms by affecting the expression of the antimicrobial peptide LL-37.^[17,18]^

Based on this, this study draws on nationally representative data from NHANES to systematically analyze the intakes of nutrients such as calcium, fat, and vitamin D. It also examines their associations with ECC in conjunction with factors including breastfeeding history, age, and ethnicity. Innovatively, it employs restricted cubic spline (RCS) regression to explore the nonlinear effects of calcium intake, aiming to provide a scientific basis for the early prevention and precise intervention of ECC.

## 2. Materials and methods

### 2.1. Research subject

The data of this study were derived from NHANES, which is conducted by the National Center for Health Statistics and the Centers for Disease Control and Prevention of the United States (U.S.). As a cross-sectional survey, it aims to obtain nationally representative data on the U.S. civilian population. This study included all respondents from the publicly available data files of NHANES for 3 cycles: 2009 to 2010, 2011 to 2012, and 2013 to 2014. The reason for limiting the study period to 2014 is that the data collection methods and diagnostic criteria for ECC in NHANES remained relatively consistent during these 3 cycles, which could ensure the comparability and reliability of the data. Additionally, using data from these 3 consecutive cycles could provide a sufficient sample size for our analysis. The data collection protocols were approved by the National Center for Health Statistics Institutional Review Board, and all survey participants signed informed consent forms prior to undergoing interviews and examinations.

### 2.2. Patient grouping

Taking all included respondents as the overall study subjects, subsequent statistical analysis methods were employed to explore the associations between factors such as nutrition, diet, and ECC. We also conducted sensitivity analyses to assess the robustness of our findings. Moreover, considering the potential impact of fluoride use on caries prevention, we adjusted for fluoride use in our statistical models. The information on fluoride use was collected from the NHANES questionnaire, which asked about the frequency and type of fluoride-containing products used, such as toothpaste and mouthwash.

### 2.3. Data collection

This study collected various types of information using a standardized questionnaire, covering age, gender, ethnicity, body mass index (BMI), energy intake, intake of various nutrients such as calcium, protein, and dietary fiber, as well as information including whether the child was breastfed and the first type of milk fed. For missing values in the data, we used the multiple imputation method in R software for imputation. Multiple imputation is a more robust approach compared to single imputation as it accounts for the uncertainty associated with missing data by creating multiple imputed datasets and then combining the results from these datasets. This helps to reduce bias and improve the accuracy of our statistical analyses. For missing values in the data, the interpolation method in R software was used for imputation. The diagnosis of caries was not solely based on the assessment results from the self-reported questionnaire in NHANES’ multiple-choice question (oral health multiple-choice question) asking “Do you have caries?” We also considered other relevant information from the NHANES oral health examination, such as the presence of decayed, missing, or filled teeth scores. However, due to the limitations of the available data, the self-reported questionnaire still played a significant role in our caries diagnosis. We acknowledge that self-reported data may have some limitations in terms of accuracy, but it is a commonly used method in large - scale epidemiological studies.

### 2.4. Statistical methods

All statistical analyses were performed in accordance with the guidelines of the U.S. Centers for Disease Control and Prevention, with sampling weights applied to obtain nationally representative prevalence estimates for the noninstitutionalized U.S. population. Continuous variables were presented as mean ± standard deviation, and categorical variables as numbers and percentages. Normally distributed quantitative data were described as mean ± standard deviation (x ± s), with the *t*-test used to assess intergroup differences. Non-normally distributed data were described as median and interquartile range (M (P25, P75)), and intergroup differences were compared using the weighted *t*-test (for continuous variables) or weighted chi-square test (for categorical variables). Forest plots were used to explore the effects of various factors on caries; multivariate logistic regression was employed to analyze the impacts of nutritional factors, age, ethnicity, etc on ECC; bivariate analysis was conducted to examine the correlations between nutritional factors or trace elements and ECC; and RCS regression was used to investigate the nonlinear relationship between calcium and caries. A *P* value < .05 was considered statistically significant for determining differences.

## 3. Result

### 3.1. Baseline characteristics

A total of 2564 participants’ survey data were collected and included in the final analysis of this study. After grouping by the presence or absence of caries, there were 2217 caries-free individuals and 347 with caries. Preliminary analysis showed significant differences in multiple clinical variables between the 2 groups.

Regarding demographic characteristics and gender distribution among children with caries, there were 169 males (48.7%) and 179 females (51.3%), with no significant gender ratio difference (*P* = .659). There was a significant difference in ethnic distribution (*P* < .01): Mexican Americans accounted for 29.1% in the caries group, which was higher than 22.3% in the caries-free group, while non-Hispanic Whites accounted for 17.0% in the caries group, lower than 28.9% in the caries-free group. In terms of educational level, the vast majority of participants in both groups (over 97% in each) had never received education or only attended kindergarten, with no significant difference between groups (*P* = .788). In terms of feeding status, breastfeeding was significantly associated with the occurrence of caries (*P* = .002): 213 individuals (61.4%) in the ever-breastfed group had caries, which was significantly higher than 134 individuals (38.6%) in the non-breastfed group. However, there was no significant difference in the type of milk first fed (whole milk, low-fat milk, etc) between the 2 groups (*P* = .283). Age distribution showed that children with caries were mainly aged 4 years and above. The proportions of children with caries at 2, 3, 4, 5, and 6 years old were 6.3%, 15.9%, 24.8%, 23.6%, and 29.4%, respectively, with an extremely significant difference in age (*P* < .01).

In terms of nutrient intake, there were significant differences in the intake of energy (*P* = .039), fat (g/day) (*P* = .022), vitamin D (*P* = .029), and calcium (*P* = .021) between the caries group and the caries-free group. The average energy intake in the caries group was slightly higher than that in the caries-free group; fat intake was higher in the caries group, while the average intake of vitamin D and calcium in the caries group was lower than that in the caries-free group. There were no significant differences between the 2 groups in the intake of other macronutrients (such as total sugar, dietary fiber, and protein), vitamins, and minerals. In addition, the mean BMI of children in both groups was 16.10, with no statistically significant difference in BMI between the 2 groups (*P* = .823) (Table 1).

**Table 1:** Baseline characteristics.

Variable		ECC		*P* value
		No (n = 2217)	Yes (n = 347)	
Age	2 years old	626 (28.2%)	22 (6.3%)	< .01
	3years old	404 (18.2%)	55 (15.9%)	
	4 years old	174 (18.8%)	86 (24.8%)	
	5 years old	343 (15.5%)	82 (23.6%)	
	6 years old	427 (19.3%)	102 (29.4%)	
Gender	Male	1108 (50.0%)	169 (48.7%)	.659
	Female	1109 (50.0%)	179 (51.3%)	
Race	Mexican American	494 (22.3%)	101 (29.1%)	< .01
	Non-Hispanic White	640 (28.9%)	59 (17.0%)	
	Non-Hispanic Black	507 (22.9%)	99 (28.5%)	
	Other Hispanic	265 (12.0%)	44 (12.7%)	
	Other Race	311 (14.0%)	44 (12.7%)	
Education level	Never /Only attended kindergarten	2153 (97.1%)	337 (97.1%)	.788
	Grade 1	61 (2.8%)	9 (2.6%)	
	Grade 2	3 (0.1%)	1 (0.3%)	
Breastfed	No	670 (30.2%)	134 (38.6%)	.002
	Yes	1547 (69.8%)	213 (61.4%)	
First cow’s milk feeding	whole milk	1751 (79.0%)	258 (74.4%)	.283
	2% milk	388 (17.5%)	75 (21.6%)	
	1% milk	28 (1.3%)	7 (2.0%)	
	skim milk	10 (0.5%)	3 (0.9%)	
	soy milk	40 (1.8%)	4 (1.2%)	
Energy		1494.00 (1181.50–1889.50)	1560.00 (1222.00–1986.00)	.039
Total Sugars		99.60 (72.49–132.72)	100.88 (73.67–136.03)	.299
Dietary Fiber		10.90 (7.60–15.20)	11.40 (8.20–15.50)	.269
Macronutrients	Protein	52.71 (39.27–68.55)	54.25 (40.51–68.27)	.342
	Carbohydrates	205.39 (158.25–262.85)	217.31 (158.61–271.34)	.084
	Fat	52.96 (38.32–70.76)	55.77 (42.21–72.89)	.022
Vitamin	VA	506.00 (325.50–732.50)	472.00 (291.00–713.00)	.057
	VC	65.20 (30.55–114.75)	69.80 (31.50–125.90)	.230
	VD	5.60 (3.30–8.60)	5.00 (3.00–8.00)	.029
Minerals	Calcium	893.00 (626.00–1243.50)	838.00 (571.00–1156.00)	.021
	Phosphorus	1055.00 (789.50–1345.00)	1027.00 (767.00–1319.00)	.270
	Zinc	7.66 (5.44–10.36)	7.99 (5.46–10.77)	.259
	Iron	10.58 (7.35–14,366)	11.36 (7.71–15.81)	.098
BMI		16.10 (15.20–17.20)	16.10 (15.20–17.52)	.823

BMI = body mass index, ECC = early childhood caries, n = number of individuals, VA = vitamin A, VC = vitamin C, VD = vitamin D.

### 3.2. Forest plot illustrating the impact of various factors on ECC disease

**Figure 1.**
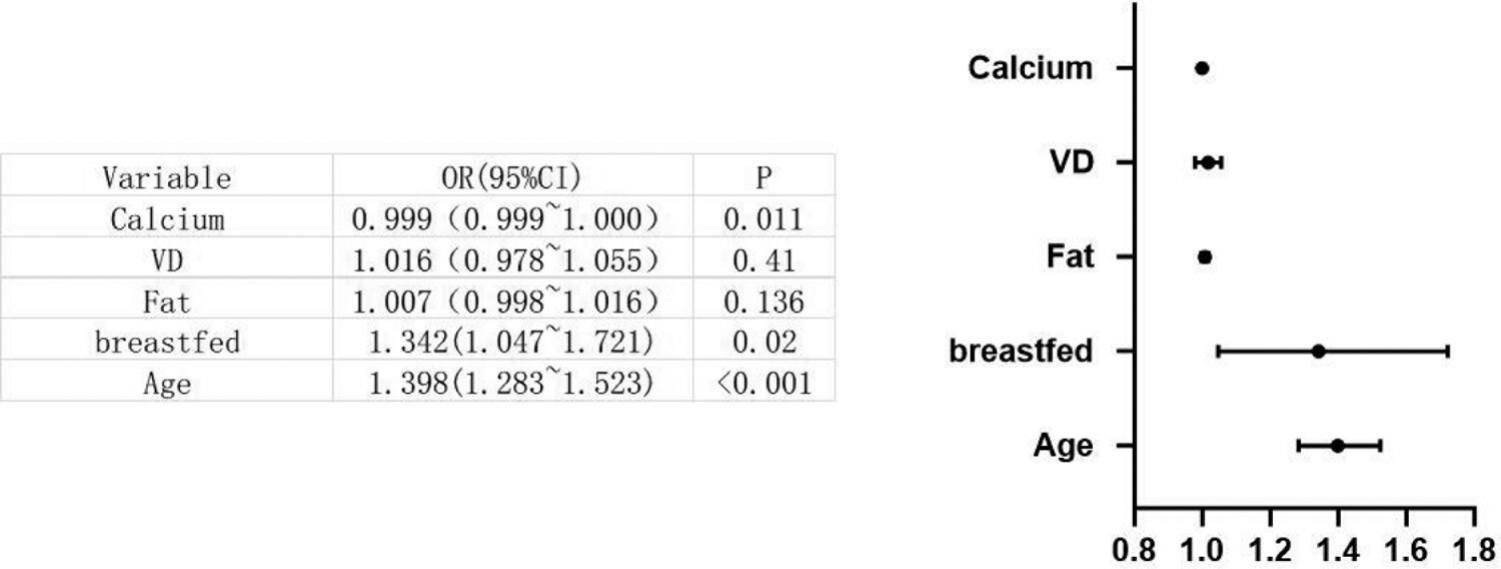
Forest plot showing the associations between nutritional, demographic, and feeding factors and the risk of ECC. This forest plot presents the OR with 95% CI and *P* values for 5 variables (calcium intake, vitamin D intake, total fat intake, breastfeeding history, and age) in relation to ECC among children aged 2 to 6 years. Significant associations were found for calcium intake (*P* = .011), lack of breastfeeding history (*P* = .020), and increasing age (*P* < .01). No statistically significant association was observed for vitamin D or total fat intake. CI = confidence interval, ECC = early childhood caries, OR = odds ratio, VD = vitamin D.

A forest plot was used to display the associations between 5 variables (calcium, vitamin D, total fat, history of breastfeeding, and age) and their impacts on dental caries in young children. Each variable had a corresponding odds ratio (OR) with its 95% confidence interval (CI) and the respective *P* value. An analysis of the associations between calcium, vitamin D, total fat, history of breastfeeding, age, and the impact on dental caries in young children showed that there was a certain degree of correlation between calcium (OR = 0.999, 95% CI: 0.999–1.000, *P* = .011), no history of breastfeeding (OR = 1.342, 95% CI: 1.047–1.721, *P* = .02), age (OR = 1.398, 95% CI: 1.283–1.523, *P* < .001), vitamin D (OR = 1.016, 95% CI: 0.978–1.055, *P* = .41), and the impact on dental caries in young children, and all of them had a significant impact on dental caries in young children (*P* = .011, *P* = .02, *P* < .01). In contrast, although total fat (OR = 1.007, 95% CI: 0.998–1.016, *P* = .136) showed intergroup differences in baseline characteristics, the forest plot analysis indicated that their associations with dental caries in young children did not reach a significant level (Fig. 1).

### 3.3. Multivariate logistic regression analysis of the effects of various risk factors on ECC

The results of the multivariate logistic regression analysis showed that age and race were significant factors affecting ECC (*P* < .05). Compared with 2-year-old children, the risk of caries was significantly higher in children aged 3 to 6, with 5-year-old children having the highest risk (OR = 6.49, 95% CI: 3.955–10.663). Regarding race, using Mexican Americans as a reference, non-Hispanic Black and other racial populations had lower risks of caries (ORs were 0.45 and 0.65, respectively). Total fat intake and vitamin D levels did not show significant associations (*P* > .05) (Table 2).

**Table 2:** Multivariate logistic regression model for ECC.

	B	SE	Wals	OR (95% CI)	*P*
Age			65.656		< .001
3 years old	1.366	0.262	27.225	3.92 (2.346, 6.547)	< .001
4 years old	1.736	0.249	48.61	5.676 (3.484, 9.248)	< .001
5 years old	1.871	0.253	54.692	6.494 (3.955, 10.663)	< .001
6 years old	1.843	0.248	55.279	6.319 (3.887, 10.272)	< .001
Race			24.735		< .001
Non-Hispanic White	-0.248	0.2	1.527	0.781 (0.527, 1.156)	.217
Non-Hispanic Black	-0.794	0.178	19.902	0.452 (0.319, 0.641)	< .001
Other Hispanic	-0.052	0.162	0.104	0.949 (0.691, 1.303)	.748
Other Race	-0.424	0.199	4.561	0.654 (0.443, 0.966)	.033
Fat	0.004	0.002	2.822	1.004 (0.999, 1.008)	.093
VD	-0.02	0.015	1.687	0.98 (0.952, 1.01)	.194

The variance inflation factor (VIF) of each variable in the model is mostly between 1 and 1.5, indicating that there is no serious multicollinearity issue in the model.

B = beta coefficient, CI = confidence interval, ECC = early childhood caries, OR = odds ratio, SE= standard error, VD = vitamin D.

### 3.4. Correlation analysis of the impact of various risk factors on ECC

Based on the results of the correlation analysis between dental caries in young children and various factors, we found that age, total energy, and total fat were significantly positively correlated with dental caries in young children (*P* < .01, *P* = .006, *P* = .002). This indicates that age, total energy, and total fat have a significant impact on the occurrence of dental caries in young children. There was a positive correlation between calcium and dental caries in young children (*P* = .058). Although the *P* value did not reach the .05 significance level, it was close to this value, so the potential association between calcium and dental caries in young children still deserves attention. Other factors (vitamin D, ethnicity) showed no significant correlation with dental caries in young children (*P* > .05). It is worth noting that although the correlation of some variables was not significant, they may still affect dental caries in young children to a certain extent. However, these speculations need to be verified by further studies. In general, age, total energy, and total fat are important related factors affecting dental caries in young children, and future studies can further explore their mechanism of action (Table 3).

**Table 3:** Correlation analysis between ECC and various factors.

Variable	Correlation coefficient	*P* value
Age	0.173**	< .001
Race	−0.29	.145
VD	−0.035	.08
Calcium	−0.038	.058
Energy	−0.054**	.006
Fat	0.045*	.022

* When the confidence level (double test) is 0.05, the correlation is significant.

** When the confidence level (double test) is 0.001, the correlation is significant.

ECC = early childhood caries, VD = vitamin D.

### 3.5. RCS regression was used to explore the nonlinear relationship between calcium and ECC

**Figure 2.**
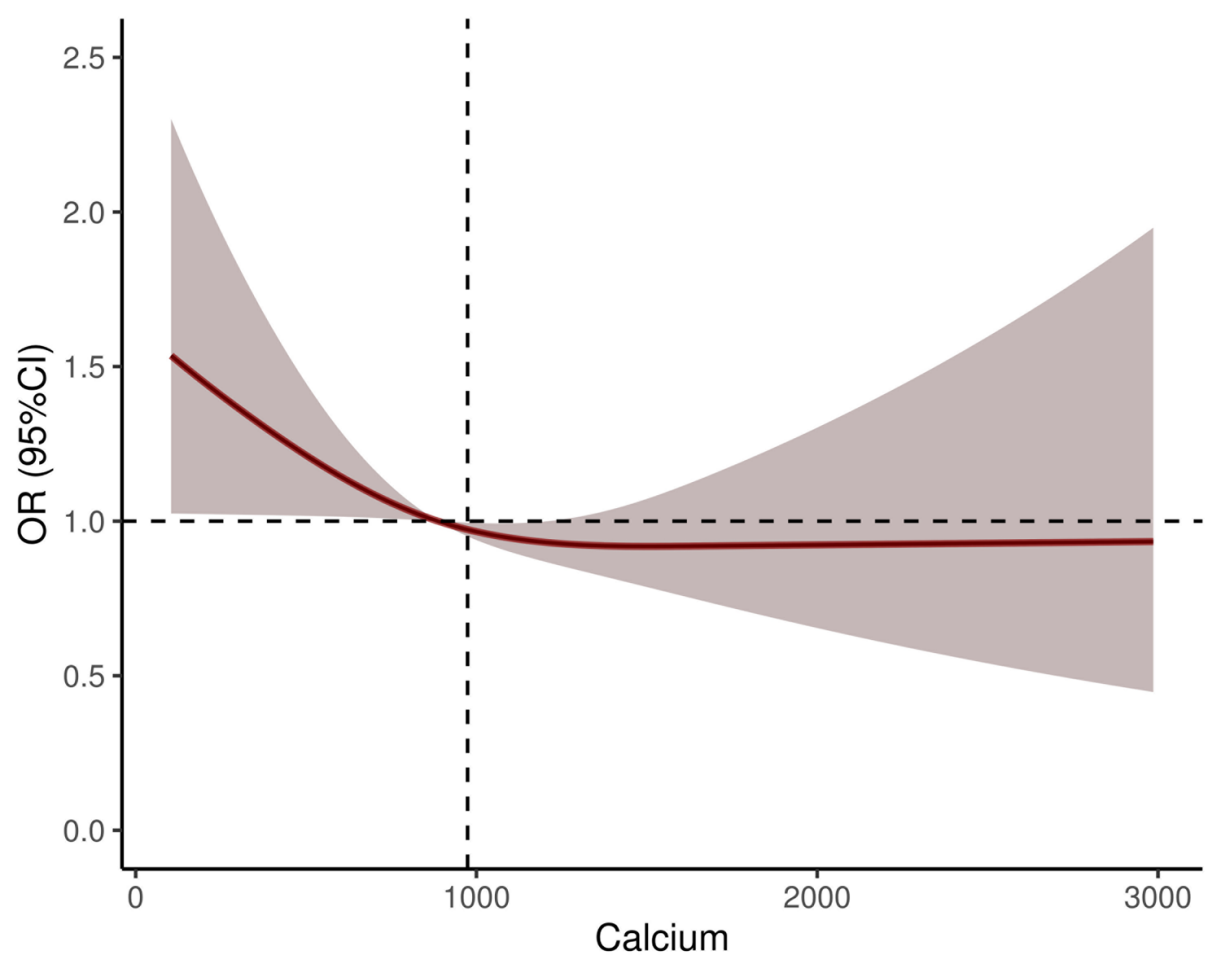
RCS regression model depicting the nonlinear relationship between calcium intake and the risk of ECC. The RCS regression curve illustrates a nonlinear association between daily calcium intake and the odds of ECC, after adjusting for confounding variables. A significant nonlinear trend was observed (*P* for nonlinearity = .011), with a turning point at 1000 mg/day. Below this threshold, increasing calcium intake was associated with a decreasing risk of ECC; above 1000 mg/day, the risk tended to plateau. The solid line indicates the estimated OR, and the shaded area represents the 95% CI. CI = confidence interval, ECC = early childhood caries, OR = odds ratio, RCS = restricted cubic spline.

In the RCS regression analysis, after adjustment for potential covariates, a significant nonlinear relationship between calcium and ECC was detected (*P* value for the nonlinearity test < .011) (Fig. 1). With the increase in calcium intake, the OR of dental caries in young children showed a decreasing trend. The RCS analysis identified a significant inflection point: the inflection point was at 1000. When calcium intake was higher than 1000, the disease risk in young children gradually stabilized; at high calcium levels, the OR remained stable, and the CI was also relatively stable. This may be due to the influence of other factors (Fig. 2).

## 4. Discussion

This study, based on the NHANES database, systematically analyzed the associations between nutritional, feeding, and demographic factors and ECC among 2564 children aged 2 to 6 years. Through multidimensional statistical methods (forest plots, multiple linear regression, correlation analysis, and RCS regression), it revealed significant effects of factors such as calcium intake, breastfeeding, and age on ECC. Beyond validating traditional risk factors, this study provides novel insights into the pathological mechanisms linking diet, microbial activity, and caries development, offering a scientific foundation for targeted interventions. The study not only validated traditional risk factors but also provided new insights into the pathological mechanisms linking diet, microbial activity, and the development of dental caries, laying a scientific foundation for targeted interventions.

Baseline analysis showed that the ECC group and the non-ECC group had significant differences in ethnicity, breastfeeding history, age, and intake levels of calcium, vitamin D, and fat. Mexican Americans had a higher caries rate (29.1 vs 22.3%), which may be related to differences in cultural dietary habits or oral health care. Previous studies have indicated that this population has a higher proportion of refined carbohydrates in their diet, and such a dietary pattern can significantly increase the abundance of *S. mutans* and *Lactobacillus acidophilus* in the oral cavity.^[19,20]^

The prevalence of dental caries in breastfed infants and young children was significantly higher than that in non-breastfed ones (61.4 vs 38.6%). This phenomenon may be closely related to the acidification of the oral environment caused by frequent nighttime breastfeeding behavior.^[10]^ Although breast milk itself contains antibacterial components such as lactoferrin, nighttime breastfeeding may increase the risk of caries through the following mechanisms: First, it prolongs exposure to an acidic oral environment, which selectively promotes the proliferation of acid-resistant microorganisms; second, it reduces salivary flow rate and buffering capacity, thereby exacerbating dysbiosis of the oral microbial community.^[15]^ From a clinical perspective, these findings suggest that breastfeeding practices, particularly nighttime feeding, should be complemented with enhanced oral hygiene measures to mitigate caries risk. In terms of age distribution, the risk of ECC showed a significant upward trend with increasing age, with the prevalence rate reaching 29.4% among 6-year-old children. This characteristic is consistent with the law of cumulative effect of deciduous tooth caries, suggesting that the period after 3 years of age is a critical period for caries prevention and control.^[21,22]^

Results of the nutritional factor analysis showed that insufficient calcium intake (838 mg in the caries group vs 893 mg in the non-caries group) was significantly positively correlated with the risk of ECC (*P* = .021).^[23]^ RCS regression analysis further revealed a nonlinear association between calcium intake and ECC: when calcium intake was < 1000 mg, the risk of ECC decreased with the increase in intake; after exceeding this threshold, the risk tended to stabilize. Biologically, this threshold effect may be explained by calcium ions acting as signaling molecules that regulate cariogenic bacterial biofilm formation and by maintaining salivary calcium-phosphate saturation, which promotes enamel remineralization. This indicates that a daily calcium intake of 1000 mg may serve as the threshold for reducing the risk of ECC.^[24]^ This threshold effect may be explained by how calcium ions influence oral microecology: by acting as signaling molecules that regulate the formation of cariogenic bacterial biofilms,^[25]^ and by maintaining salivary calcium-phosphate saturation, which promotes a remineralization-friendly microenvironment on the enamel surface.^[26,27]^ This finding is consistent with the theory regarding the mineralization requirements of bones and enamel; however, consideration should be given to the potential confounding effect of calcium sources (e.g., dairy products may concurrently provide protective proteins).^[28,29]^

The association of vitamin D did not reach statistical significance (*P* = .105). This may be related to the generally low intake of vitamin D observed in the study (median: 5.6 μg) and the absence of serum 25-hydroxyvitamin D level measurements. Its potential mechanisms may involve 2 aspects. First, the distribution of vitamin D receptor gene polymorphisms varies among different populations.^[30,31]^ Second, the effect of vitamin D on the oral microbiota may be indirectly mediated by its regulation of antimicrobial peptide expression (e.g., defensins), and this regulatory process may exhibit a delayed dose-response relationship.^[32]^ Further studies are recommended to incorporate serum 25-hydroxyvitamin D measurements and microbiome sequencing to validate these findings.

The limitations of this study include: First, the diagnosis of dental caries relies on a combination of parent-reported questionnaire data and available oral health examination data (such as the decayed, missing, or filled teeth index index), which may still introduce potential misclassification bias. Future studies should combine clinical examination data to improve accuracy. Second, the cross-sectional design cannot infer causal relationships; for example, low calcium intake may reflect poor overall dietary quality rather than being directly pathogenic. Additionally, confounding factors such as oral hygiene habits and fluoride exposure were not analyzed. To enhance clinical applicability, it is recommended that future longitudinal studies be conducted, integrating dietary records with biochemical markers (e.g., serum calcium, vitamin D), and exploring the impact of breastfeeding patterns (such as the timing of nighttime weaning) on ECC.

## 5. Conclusion

This study reveals a close association between ECC and nutrition, feeding patterns, and the oral microbiome. It was found that a calcium intake of 1000 mg/day can significantly reduce the risk of ECC by regulating oral microbial balance (e.g., inhibiting the formation of *S. mutans* biofilms), while high-fat diets promote the proliferation of cariogenic microorganisms such as Bacteroidetes, thereby increasing the disease risk. Changes in the acidic environment caused by nighttime breastfeeding can lead to the dominant growth of acid-tolerant flora (e.g., *Lactobacillus*). Children aged 6 had the highest prevalence rate of ECC (29.4%), which is closely related to oral dysbiosis caused by their increased dietary autonomy. These findings provide a basis for formulating prevention strategies based on microbiome regulation. It is recommended to pay attention to oral microecological balance and optimize feeding patterns while ensuring adequate calcium intake. Future studies need to further explore the interaction mechanisms among nutrition, microorganisms, and caries.

## Author contributions

**Data curation:** Xiao-Xiao Zhang.

**Formal analysis:** Qi Sun.

**Investigation:** Tian-Ke Li.

**Methodology:** Hui-Ying Li.

**Project administration:** Shao-Cong Ma.

**Supervision:** Li-Hong Liu.

**Validation:** Tian-Ke Li.

**Visualization:** Xiao-Xiao Zhang.

**Writing – original draft:** Li-Shan Ren.

**Writing – review & editing:** Zi-Ying Ji.

## References

[1] Nadeeshani H Kudagammana ST Herath C Jayasinghe R Liyanage R. Early childhood caries and nutritional status of children: a review. Food Nutr Bull. 2023;44:249–64. 38095292 10.1177/03795721231209358

[2] Tungare S Paranjpe AG. StatPearls. 2025.

[3] Uribe SE Innes N Maldupa I. The global prevalence of early childhood caries: a systematic review with meta-analysis using the WHO diagnostic criteria. Int J Paediatr Dent. 2021;31:817–30. 33735529 10.1111/ipd.12783

[4] Wang X Ma Z Lei M. Association between early childhood caries and diet quality among Chinese children aged 2-5 years. Front Public Health. 2022;10:974419. 36568786 10.3389/fpubh.2022.974419 PMC9782538

[5] Kotha A Vemulapalli A Mandapati SR Aryal S. Prevalence, trends, and severity of early childhood caries in the United States: national health and nutritional examination survey Data 2013 to 2018. Pediatr Dent. 2022;44:261–8. 35999681

[6] Zou J Du Q Ge L. Expert consensus on early childhood caries management. Int J Oral Sci. 2022;14:35. 35835750 10.1038/s41368-022-00186-0 PMC9283525

[7] van Meijeren-van Lunteren AW Voortman T Elfrink M Wolvius EB Kragt L. Breastfeeding and childhood dental caries: results from a socially diverse birth cohort study. Caries Res. 2021;55:153–61. 33706311 10.1159/000514502 PMC8117384

[8] Sandy L Helmyati S Amalia R. Nutritional factors associated with early childhood caries: a systematic review and meta-analysis. Saudi Dent J. 2024;36:413–9. 38525179 10.1016/j.sdentj.2023.12.001 PMC10960096

[9] Ma S Ma Z Wang X. Relationship of dietary nutrients with early childhood caries and caries activity among children aged 3-5 years-a cross-sectional study. BMC Pediatr. 2024;24:506. 39112952 10.1186/s12887-024-04984-9 PMC11304563

[10] Shrestha SK Arora A Manohar N Ekanayake K Foster J. Association of breastfeeding and early childhood caries: a systematic review and meta-analysis. Nutrients. 2024;16:1355. 38732602 10.3390/nu16091355 PMC11085424

[11] Chiao C Kaye E Scott T Hayes C Garcia RI. Breastfeeding and early childhood caries: findings from the national health and nutrition examination survey, 2011 to 2018. Pediatr Dent. 2021;43:276–81. 34467843

[12] Dhull KS Dutta B Pattanaik S Gupta A Md I Wandile B. Decoding early childhood caries: a comprehensive review navigating the impact of evolving dietary trends in preschoolers. Cureus. 2024;16:e58170. 38741840 10.7759/cureus.58170 PMC11090680

[13] Marshall TA Touger-Decker R. Oral health and multimorbidity: is diet the chicken or the egg. Proc Nutr Soc. 2024:1–8. 10.1017/S0029665124004683 38742385

[14] Tian K Xiao C Chen Y. Proline-rich protein from S. mutans can perform a competitive mineralization function to enhance bacterial adhesion to teeth. Sci Rep. 2022;12:22250. 36564474 10.1038/s41598-022-26303-x PMC9789152

[15] Panchanadikar NT S A Muthu MS H S Jayakumar P Agarwal A. Breastfeeding and its association with early childhood caries - an umbrella review. J Clin Pediatr Dent. 2022;46:75–85. 35533221 10.17796/1053-4625-46.2.1

[16] Carrillo-Díaz M Ortega-Martínez AR Ruiz-Guillén A Romero-Maroto M González-Olmo MJ. Impact of breastfeeding and cosleeping on early childhood caries: a cross-sectional study. J Clin Med. 2021;10:1561. 33917683 10.3390/jcm10081561 PMC8067957

[17] Williams TL Boyle J Mittermuller BA Carrico C Schroth RJ. Association between vitamin D and dental caries in a sample of Canadian and American preschool-aged children. Nutrients. 2021;13:4465. 34960016 10.3390/nu13124465 PMC8706858

[18] DiTosto JD Caniglia EC Hinkle SN. Target trial emulation of preconception serum vitamin D status on fertility outcomes: a couples-based approach. Fertil Steril. 2025;123:300–12. 39173703 10.1016/j.fertnstert.2024.08.332 PMC11788044

[19] Luo H Moss ME Wright W Webb M Pardi V Lazorick S. Racial/ethnic disparities in preventive dental services use and dental caries among children. J Public Health Dent. 2023;83:161–8. 36883255 10.1111/jphd.12563 PMC10258156

[20] Luo H Wu B Wu Y Moss ME. Dental caries and preventive dental visits among children in the U.S.: the impact of race/ethnicity and immigration. AJPM Focus. 2024;3:100230. 38766463 10.1016/j.focus.2024.100230 PMC11099302

[21] Tang Z Xu W Zhou Z Qiao Y Zheng S Rong W. Taxonomic and functional alterations in the salivary microbiota of children with and without severe early childhood caries (S-ECC) at the age of 3. PeerJ. 2022;10:e13529. 35669952 10.7717/peerj.13529 PMC9165595

[22] Khan SY Javed F Ebadi MH Schroth RJ. Prevalence and risk factors for ECC among preschool children from India along with the need of its own CRA tool- a systematic review. J Int Soc Prev Community Dent. 2022;12:295–308. 35966917 10.4103/jispcd.JISPCD_56_22 PMC9369784

[23] Crescente CL de Sousa ET Lima-Holanda AT Steiner-Oliveira C Nobre-Dos-Santos M. Biofilm accumulation and sucrose rinse modulate calcium and fluoride bioavailability in the saliva of children with early childhood caries. Sci Rep. 2022;12:10283. 35717506 10.1038/s41598-022-14583-2 PMC9206641

[24] Zhang Q Bai X Jin H Dong N. Combined effect of dietary calcium consumption and physical activity on dental caries in children and adolescents: a study of the NHANES database. BMC Oral Health. 2024;24:281. 38419086 10.1186/s12903-024-03969-5 PMC10900671

[25] Sedghi L DiMassa V Harrington A Lynch SV Kapila YL. The oral microbiome: Role of key organisms and complex networks in oral health and disease. Periodontol 2000. 2021;87:107–31. 34463991 10.1111/prd.12393 PMC8457218

[26] Raphael S Blinkhorn A. Is there a place for Tooth Mousse in the prevention and treatment of early dental caries? A systematic review. BMC Oral Health. 2015;15:113. 26408042 10.1186/s12903-015-0095-6 PMC4583988

[27] Singal K Sharda S Gupta A. Effectiveness-of calcium phosphate derivative agents on the prevention and remineralization of caries among children- a systematic review & meta-analysis of randomized controlled trials. J Evid Based Dent Pract. 2022;22:101746. 36162884 10.1016/j.jebdp.2022.101746

[28] Gil-Bona A Bidlack FB. Tooth enamel and its dynamic protein matrix. Int J Mol Sci . 2020;21:4458. 32585904 10.3390/ijms21124458 PMC7352428

[29] Tay N Gan H de Sousa FB. Improved mineralization of dental enamel by electrokinetic delivery of F(-) and Ca(2+) ions. Sci Rep. 2023;13:516. 36627315 10.1038/s41598-022-26423-4 PMC9832158

[30] Hung M Mohajeri A Sadri M Khodabandeh E Zeitoun I Lipsky MS. The association of vitamin D levels and dental caries in older adults: a cross-sectional study. Nutrients. 2024;16:2307. 39064749 10.3390/nu16142307 PMC11279458

[31] Karras SN Dursun E Alaylioğlu M. Investigating the role of functional polymorphism of maternal and neonatal vitamin D binding protein in the context of 25-hydroxyvitamin D cutoffs as determinants of maternal-neonatal vitamin D status profiles in a sunny Mediterranean region. Nutrients. 2021;13:3082. 34578960 10.3390/nu13093082 PMC8467735

[32] Zanetta P Squarzanti DF Sorrentino R. Oral microbiota and vitamin D impact on oropharyngeal squamous cell carcinogenesis: a narrative literature review. Crit Rev Microbiol. 2021;47:224–39. 33476522 10.1080/1040841X.2021.1872487

